# Early antifungal resistance prediction based on MALDI-TOF mass spectrometry and machine learning

**DOI:** 10.1038/s41598-026-53519-y

**Published:** 2026-06-02

**Authors:** Diane Duroux, Yiqi Yang, Janko Sattler, Michael Krauthammer, Adrian Egli

**Affiliations:** 1 ETH AI Center, ETH Zurich, Zurich, Switzerland; 2 Department of Biosystems Science and Engineering, ETH Zurich, Basel, Switzerland; 3 Department of Quantitative Biomedicine, University of Zurich, Zurich, Switzerland; 4 SIB Swiss Institute of Bioinformatics, Basel, Switzerland; 5 Department of Machine Learning and Systems Biology, Max Planck Institute of Biochemistry, Martinsried, Germany; 6 Institute of Medical Microbiology, University of Zurich, Zurich, Switzerland

**Keywords:** Computational biology and bioinformatics, Diseases, Microbiology

## Abstract

Antimicrobial resistance (AMR) is a significant global health threat. Recent studies have shown that combining MALDI-TOF mass spectrometry with machine learning algorithms can accelerate AMR determination. However, these efforts have predominantly focused on bacterial pathogens. The significant morbidity, mortality, and healthcare costs associated with fungal infections highlight the need for accurate and early detection of antifungal resistance. We developed a machine learning pipeline integrating MALDI-TOF mass spectrometry data and drug features to predict antifungal resistance and identify spectral biomarkers. By leveraging the DRIAMS dataset, we included 658 pathogen spectra linked to 3,046 phenotypic antifungal resistance results across three drug classes and seven yeast species. Models were trained using categorical phenotypic antifungal susceptibility testing results as ground truth. We systematically investigated how different dimensionality reduction methods, antifungal encodings, and model types affected predictive performance using nested cross-validation. We identified that applying principal component analysis to MALDI-TOF mass spectra, and training a multi-layer perceptron yielded the highest and most stable performance for the prediction of antifungal resistance. Our method achieved an AUPRC of 0.77 across the 10 highest-performing species-drug pairs. The model demonstrated the best performance for the species-drug combinations of *Candida albicans* with micafungin, *Candida parapsilosis* with fluconazole, and *Saccharomyces cerevisiae* with itraconazole and fluconazole. By comparing established species-based guidelines, susceptibility test results, and machine learning predictions, we estimated that integrating our algorithm into antifungal selection could help avoid prescriptions to likely resistant pathogens in approximately 3 out of 10 patients for whom standard guidelines recommend such treatments.

## Introduction

Fungal infections are an increasingly significant global health threat, with an estimated 1.5 million deaths annually attributed to invasive fungal diseases^1^. These infections are particularly alarming for clinicians due to their high mortality rate, which often exceeds 50%^2^. Invasive fungal infections are becoming increasingly harder to treat due to the rising prevalence of antifungal resistance^3^. Unlike bacterial infections, for which a larger choice of antimicrobial treatments exists, options for combating fungal infections are far more limited. This limitation makes accurate prediction and management of antifungal resistance clinically important. As resistance continues to spread, the consequences are severe, resulting in longer hospitalizations, increased healthcare costs, and higher mortality rates^4,5^. Given the escalating burden of fungal infections, there is an urgent need for more rapid and precise diagnostic methods to inform and optimize treatment strategies.

The determination of appropriate antifungal treatment has traditionally relied on identifying fungal pathogens using Matrix-assisted laser desorption/ionization time-of-flight mass spectrometry (MALDI-TOF MS), followed by subculture of single colonies and determination of antifungal susceptibility testing (AFST). This conventional procedure can take up to 72 h to yield results, a delay that can be detrimental, especially for critically ill patients who require immediate and effective treatment^6^.

Recent advancements have expanded the capabilities of MALDI-TOF MS, demonstrating its potential not only to accurately identify bacterial and fungal species but also to provide early insights into antimicrobial resistance^7–13^. This dual functionality could significantly improve patient outcomes by enabling more timely and targeted therapeutic interventions^14–16^. The advantages of MALDI-TOF MS in clinical settings include rapid analysis times, cost-effectiveness, and minimal sample preparation requirements, making it well-suited for routine use^17^. Moreover, the integration of MALDI-TOF MS with advanced data analysis techniques, such as machine learning, has further enhanced its potential^11–13,18^. These innovations allow for the development of predictive models that can anticipate resistance patterns based on MALDI-TOF MS, potentially increasing the speed and precision of antifungal treatment decisions.

Despite the potential of MALDI-TOF MS in antimicrobial resistance prediction, significant challenges remain. While this technology has been extensively explored for bacterial infections to predict antimicrobial resistance^19–22^, its application to antifungal resistance remains underdeveloped. The lower prevalence of fungal infections has led to smaller datasets^18,23^, limiting the effectiveness of machine learning models that rely on large, diverse data. Additionally, most existing MALDI-TOF MS-based antifungal susceptibility approaches require incubating fungal strains with antifungal drugs for hours to detect proteomic changes^8,24^. While informative, these methods demand extended experimental setups and are time consuming due to the incubation with the drug, reducing their clinical practicality in urgent settings. In contrast, our approach aims to leverage MALDI-TOF spectral profiles already routinely acquired in diagnostic laboratories.

Furthermore, recent research has demonstrated that antimicrobial resistance prediction using MALDI-TOF MS can significantly benefit from large-scale deep learning models, which thrive on vast amounts of data^12,13^. However, whether these findings extend to antifungal resistance prediction remains to be explored. The advance in drug molecular representation^25,26^, i.e. encoding molecules in machine-readable formats, could also enhance performance by leveraging more efficient representations. This underscores the importance of exploring state-of-the-art machine learning approaches to push the boundaries of antifungal resistance prediction and improve predictive accuracy.

The goal of this project is to address these gaps by developing a machine learning pipeline that integrates mass spectral data with drug chemical features to predict antifungal resistance and identify the peaks contributing to the prediction. Our model will produce a binary classification indicating whether a pathogen is resistant or susceptible to a specific antifungal drug (intermediate category was not observed in the dataset). To evaluate the performance of our model, we will compare its predictions against phenotypic antifungal susceptibility testing from ISO accredited diagnostic microbiology laboratories. Additionally, to assess its potential impact on treatment selection, we will compare its predictions with species-based hospital guidelines from the University Hospital Zurich. By integrating mass spectrometry data and drug molecular structure information with advanced machine learning, this project aims to assess the diagnostic performance of MALDI-TOF MS with ML against phenotypic methods for rapid AFST.

## Materials and methods

The proposed method is intended as an early resistance warning system. Using MALDI-TOF MS data available at species identification, it aims to flag isolates likely to be resistant to guideline-recommended first-line antifungals, prompting consideration of alternative treatment. In the absence of a resistance signal, the system would not alter standard care.

### Data and pre-processing

The output to predict is a binary label for each pathogen–antifungal combination, indicating whether the pathogen is resistant or susceptible to an antifungal. The associated input for each combination consists of a MALDI-TOF mass spectrum of the pathogen and the molecular representation of the corresponding antifungal. We used the DRIAMS dataset^27^ (DOI 10.5061/dryad.bzkh1899q), containing the MALDI-TOF mass spectra of pathogens and associated antimicrobial susceptibility phenotypes.

MALDI-TOF mass spectra were acquired at four microbiological laboratories in Switzerland that provide routine diagnostic services for hospitals and private practices from 2015 to 2018. Only DRIAMS-A (University Hospital Basel) and DRIAMS-C (Canton Hospital Aarau) contained fungal data. The species of each mass spectrum was identified using the Microflex Biotyper Database included in the flexControl Software (Bruker Daltonics flexControl v3.4).

DRIAMS-C was excluded from the analyses as the pathogens were only associated with susceptible profiles, for any drug. Species-drug combinations with intrinsic resistance, as indicated by EUCAST guidelines, were excluded from the dataset. This only concerned *Candida krusei* and fluconazole (11 instances). Observations involving caspofungin associated with *Candida albicans*, *Candida glabrata*, *C. krusei*, *Candida parapsilosis*, and *Candida tropicalis* were excluded, as EUCAST guidelines recommend inferring its susceptibility from anidulafungin and micafungin. Species-drug pairs only associated with one outcome (either resistant or susceptible) were removed as the model could not learn from them. Supplementary Fig. 1 presents an overview of data exclusion steps and associated number of pathogens mass spectra and phenotypic antifungal resistance outcomes. After pre-processing, the dataset contained 658 unique pathogen spectra with 3,046 antimicrobial resistance phenotypes, corresponding to 22 pathogen-drug combinations.

We used the spectral representation defined in Weis et al.^28^, pre-processed using the R package MaldiQuant58 v1.19^29^. Namely the measured intensity was transformed to stabilize the variance, and smoothed. An estimate of the baseline was removed, the intensity was calibrated, and the spectra were trimmed to values in a 2,000–20,000 m/z range. Intensity measurements were binned using a bin size of 3 m/z units. Hence, each sample was represented by a vector of 6,000 features describing the sample mass-to-charge ratio and associated intensity.

### Pathogen mass spectra dimensionality reduction

Because the number of observations is smaller than the number of variables, we evaluated three dimensionality reduction techniques of the mass spectra and compared them both against each other and against a baseline model with no dimensionality reduction. The first approach involved applying principal component analysis (PCA) to mass spectra features. To determine the optimal number of principal components, we tested a range of explained variance thresholds (0.50, 0.75, 0.85, 0.95, and 0.99). Each threshold represents the proportion of the total variance in the original data that we aimed to retain. For each threshold, we identified the minimum number of components required to reach the target level of explained variance and evaluated the downstream performance accordingly. This allowed us to balance dimensionality reduction with information retention in the feature space.

The second approach applied a masked autoencoder (MAE). An autoencoder is a type of artificial neural network that learns to compress data into a smaller representation and then reconstruct it as accurately as possible. It has two parts: an encoder that reduces the data into a compact form, and a decoder that tries to rebuild the original data from that compact form. Unlike traditional autoencoders that compress and reconstruct input data in full, the MAE randomly masks portions of each spectrum (i.e., specific mass spectra bins) and learns to reconstruct the missing values. This encourages the model to learn meaningful patterns and dependencies within the data, potentially improving its ability to predict antifungal resistance. By masking different parts of each spectrum during training, each sample can be augmented multiple times, increasing the model’s exposure to variation in the data. This approach increases robustness and flexibility compared to standard autoencoders. We tested several MAE configurations, including different encoding dimensions (64, 128, 512), numbers of augmented copies per sample (5, 10), ranges for the proportion of masked bins (0.2–0.5 and 0.3–0.6), and batch sizes (128, 256).

The third approach focused on interpretability and used mutual information (MI) to identify the most relevant features for predicting whether a pathogen is resistant or susceptible to a given antifungal. Instead of creating new composite variables, as in PCA or autoencoders, this method selects the mass spectral bins (e.g., top 64, 128, or 512) that have the strongest statistical relationship with the outcome. As a result, the features used by the model remain tied to the original spectra, making them easier to interpret and potentially more useful for biological insights. We compared the performance of models trained on these three transformed datasets against those trained on the original, untransformed data.

These approaches were evaluated alongside the original, untransformed spectra (i.e., no dimensionality reduction), which served as a baseline and as one of the candidate representations in our comparative analysis.

### Antimicrobial embeddings

Some resistance mechanisms are shared across different species, which suggests that training models on data encompassing all species and drugs may enhance knowledge transfer and improve generalizability. To test this hypothesis, we compared several modeling strategies: one model trained on data from all species and drugs together, and other models trained separately by species, by drug, or by specific species-drug combinations. To build models that can learn across multiple drugs, it is not enough to only input information about the pathogen. The input also needs to contain information about the drug itself. Including the molecular information of a drug as part of the input can help the model understand relationships between different drugs. For example, if two drugs are chemically closely related, the pathogen may react to them in similar ways. It also means the model can learn more effectively from a larger and more diverse dataset.

We evaluated two different molecular representations of antifungal drugs for computational analysis. The first approach used the well-established Molecular ACCess Systems (MACCS) keys fingerprints^30^, which summarizes the presence or absence of 166 specific chemical features in each molecule. The second method involved advanced molecular embeddings based on a deep learning model called MoLFormer^31^. This model was trained to learn patterns in molecular structures by analyzing large collections of chemical formulas (SMILES strings) from public databases like ZINC and PubChem. It is based on an algorithm originally developed for language processing (a transformer encoder model using rotary positional embeddings), allowing it to capture complex chemical features and relationships. By employing both traditional molecular fingerprints (166 variables) and advanced transformer-based embeddings (768 variables), our objective was to evaluate the benefits and drawbacks of using interpretable but simple drug encodings compared to advanced but less interpretable features.

This resulted in a total of eight input configurations for model training: for the two antimicrobial encodings (MACCS and MoLFormer), we evaluated four spectra representations, namely original data, PCA on MS, masked autoencoder on MS, and mutual information feature selection.

**Table 1:** Summary of datasets. The training set contains 70% of the total observations, and the test set contains 30%.

	Number of antifungal resistance phenotypes	Number of species-drug combinations	Number of unique pathogen spectra	Number of unique species	Number of unique drugs	Proportion of resistant observations
Training set	2123	22	460	7	7	0.18
Test set	923	22	198	7	7	0.16

### Model architecture

Our research question was framed as a binary classification problem. The sample-drug pairs were categorized into resistant and susceptible classes based on EUCAST and CLSI recommendations as in Weis et al.^32^.

**Fig. 1:**
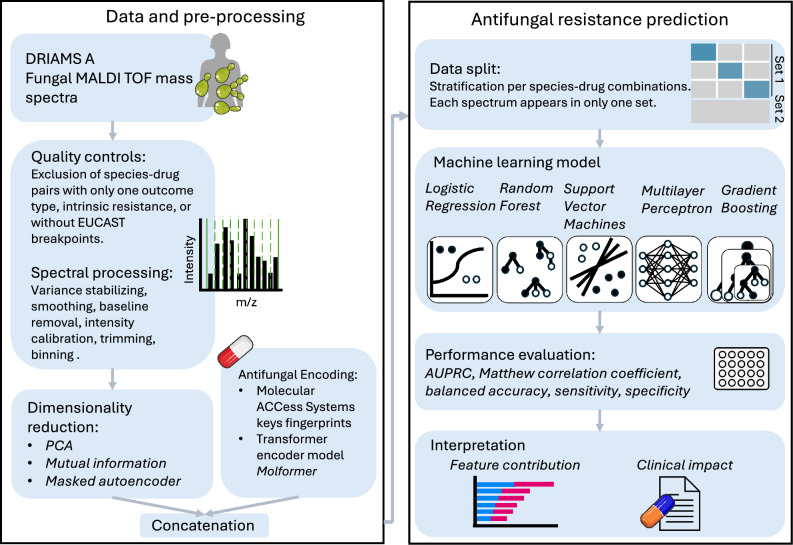
Early antifungal resistance prediction pipeline. Fungal mass spectra were selected from DRIAMS-A, a dataset derived from patient samples at University Hospital Basel. In this dataset, pathogens were cultured, their MALDI-TOF mass spectra and resistance profiles were determined, and corresponding entries were matched. Species-drug pairs associated with only one outcome (resistant or susceptible), species with intrinsic resistance to certain antifungals were excluded. Observations involving caspofungin and *Candida* spp. were excluded too, as EUCAST guidelines recommend inferring its susceptibility from anidulafungin and micafungin. Mass spectra were pre-processed and binned following Weis et al.^32^. Three dimensionality reduction techniques for spectra and two antifungal encodings were compared. The dataset was split into a training set (70%) and a test set (30%). Nested cross-validation was performed on the training set to determine the optimal preprocessing methods, machine learning models, and hyperparameters. Five machine learning approaches were evaluated. After identifying the best combination, the optimal model was applied to the test set. Model performance was assessed by comparing predictions to susceptibility test outcomes. Feature contribution analysis identified spectral peaks associated with resistance. Predictions were further compared to species-based hospital guidelines to assess the clinical impact of the tool.

To classify antifungal resistance, we evaluated five different machine learning algorithms for each of the eight input configurations (Fig. 1): logistic regression, support vector machines (SVM), random forests (RF), gradient boosting classifier (GBC), and multilayer perceptron (MLP). For each method, we conducted a hyperparameter grid search, systematically testing all possible combinations within a predefined set of parameter values, to identify the combination that resulted in the best classification performance. More details on the parameters can be found in the Supplementary Section 1. This selection covers a wide spectrum of modern machine learning techniques, ranging from classical statistical methods to deep learning algorithms, ensuring a strong evaluation of predictive performance across different methodologies. As a reference baseline configuration, we used raw pathogen mass spectra without dimensionality reduction, combined with MACCS drug encoding and logistic regression. This baseline serves as a comparator to quantify the added value of more advanced dimensionality reduction techniques and machine learning models. In total, 40 machine learning configurations were evaluated, comprising 8 input configurations combined with 5 machine learning models.

### Performance assessment

The observations were primarily divided into two sets (70% in the training set, and 30% in the test set). Stratification based on fungal species and drug combination was applied. We ensured that samples from the same fungal spectrum were included in either the training or the test set, but not both, to prevent information leakage and ensure unbiased performance evaluation. To identify the best configuration—i.e., the optimal combination of drug encoding, spectra representation, and machine learning model—and to perform hyperparameter tuning, we employed three-fold nested cross-validation within the training set, with the same stratification criteria described above. Final performance are reported on the test set. Hence, “Combination of dimensionality reduction and neural networks yield the highest antifungal prediction performance” and “Unified species-and-drug-agnostic modeling matches species-drug-specific performance and improves applicability” are based on the training set, while the final species-drug performance results (“Enhanced antifungal detection in *S. cerevisiae*, *C. albicans*, and *C. parapsilosis*”, “Identification of spectral peaks driving antifungal resistance”, and “Estimated clinical impact: reduced selection of antifungals associated with resistance”) are reported on a separate, held-out dataset (test set) that was not used during model selection.

To address the bias inherent in imbalanced datasets, we reported the Area under the precision-recall curve (AUPRC), the Matthews correlation coefficient (MCC), and the balanced accuracy. We also reported the Area Under the Receiver Operating Characteristic curve (AUROC), sensitivity, specificity, accuracy, very major error, major error, number of true positives, true negatives, false positives and false negatives. The hyperparameter search was optimized for MCC. The MCC provides a balanced measure of binary prediction performance, even in the presence of class imbalance. It ranges from -1 (total disagreement) to 1 (perfect prediction). A crucial aspect of our evaluation is the granularity of performance assessment. We focused on the species-drug combination level, as aggregated metrics can mask poor performance in specific pairs, and particularly given the over-representation of *C. albicans*, which can skew overall results. Thus, we computed and reported metrics at the species-drug pair level. To identify the optimal model configuration (dimensionality reduction, drug embedding, and ML model), we averaged these pair-level metrics, aiming to select a single best configuration. Additionally, recognizing the value of strong performance in specific pairs over uniform moderate accuracy, we also reported the average performance across the top 10 highest-performing species-drug pairs for each configuration.

### Model properties and interpretability

After identifying the best-performing configuration (see “Antimicrobial embeddings”, “Model architecture”), we conducted post hoc interpretability analyses.

In terms of interpretability, our goal was to determine whether species-specific peaks drive antifungal resistance. We investigated this question through feature importance analyses. Since PCA was part of the optimal pipeline, we examined feature importance within the PCA-transformed space. We identified the 500 spectral features contributing most to the principal components and trained an MLP model on the training set restricted to these features to assess their relevance. To quantify feature contributions and their robustness, we performed a bootstrap-based SHAP uncertainty analysis^33^. The training data were resampled with replacement 50 times, and for each bootstrap sample, an MLP classifier was retrained using the selected spectral features together with drug-related features. SHAP values were then recomputed on the same held-out test set for each selected species–drug combination. For each feature, attribution was summarized using the mean absolute SHAP value across samples and bootstrap iterations, while uncertainty was quantified using the standard deviation of SHAP values, and the 95% bootstrap interval. Because spectral variables were binned using a bin size of 3, each reported feature should be interpreted as representing the corresponding m/z value ±1. For example, if feature 3330 is reported, the corresponding m/z region should be interpreted as 3329–3331.

To evaluate the clinical relevance of our model, we assessed both the risks of incorrect antifungal resistance predictions and its potential benefits over species-based guidelines for antifungal selection. Specifically, we examined whether a patient would still receive an antifungal associated with susceptibility (based on susceptibility tests) if the model incorrectly predicts resistance and how often integrating the model into treatment decisions could prevent antifungal prescriptions associated with resistance compared to species-based guidelines. To answer these questions, we compared the model’s predictions with species-based guidelines from the University Hospital Zurich (2024), which are available upon request (https://www.usz.ch/fachbereich/infektiologie/ueber-uns/bestellen-sie-unsere-antibiotikarichtlinien/).

## Results

### Combination of dimensionality reduction and neural networks yield the highest antifungal prediction performance.

**Fig. 2:**
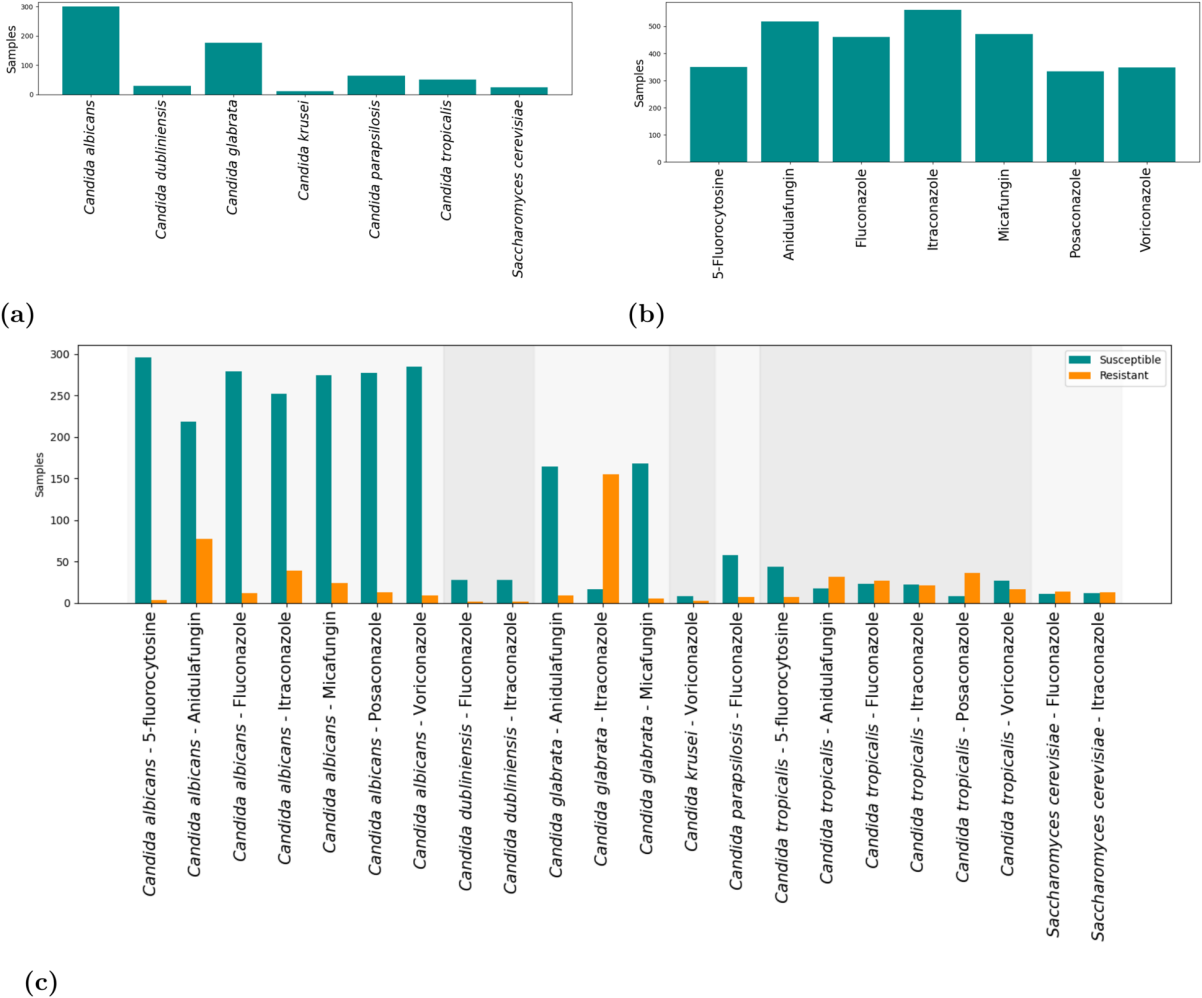
Data distribution of the fungi dataset based on (**A**) species, (**B**) drugs, and (**C**) species-drug pairs with response 0 for susceptibility and 1 for resistance.

The dataset comprises fungi from seven species, including various *Candida* species and *Saccharomyces cerevisiae* (Table 1). It includes critical fungal species identified by the WHO, such as *C. albicans* and *C. glabrata*^34^. The dataset spans seven drugs, including representatives from the azole class and echinocandins, resulting in a total of 22 species-drug combinations. The training set consists of 460 pathogen spectra, while the test set includes 198 spectra. Each observation in our dataset represents a pathogen-drug pair, as multiple drugs are tested on individual pathogen isolate. Consequently, the training set contains 2,123 antifungal resistance phenotypes, and the test set includes 923 phenotypes. Across the entire dataset (training and test sets combined), the number of samples per species varies widely (Fig. 2a), ranging from 11 for *C. krusei* to 300 for *C. albicans*. Similarly, the number of observations varies by drug, with 334 samples tested on posaconazole and 518 on anidulafungin (Fig. 2b). The majority of samples were collected in 2016 (212 samples), 2017 (268 samples), and 2018 (157 samples).

Figure 2c highlights the dataset’s class imbalance, with some species-drug pairs showing significant disparity. For instance, the *C. albicans*-5-fluorocytosine pair exhibits a stark imbalance, with 296 susceptible versus only 4 resistant outcomes. In contrast, some pairs are nearly balanced, such as *C. tropicalis*-itraconazole, with 22 susceptible and 21 resistant outcomes. Most species-drug combinations show a higher number of susceptible cases compared to resistant ones, although the opposite is observed in specific cases, such as *C. glabrata*-itraconazole and *C. tropicalis*-posaconazole. This inherent imbalance necessitated the use of evaluation metrics like balanced accuracy, AUPRC, and MCC to ensure a robust assessment of model performance.

**Fig. 3:**
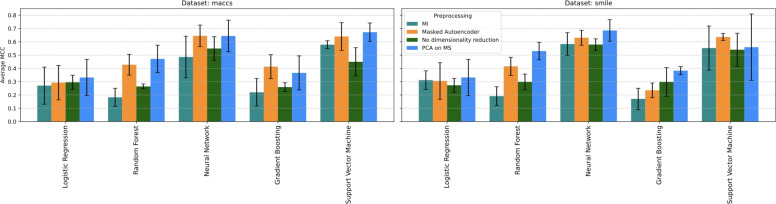
Performance evaluation (MCC) across pre-processing techniques and models for the top 10 highest-performing species-drug pairs. Error bars show the standard deviation computed via the 3-fold cross validation.

Our study systematically investigated the influence of various dimensionality reduction methods (including no dimensionality reduction as a baseline) on MALDI-TOF MS representations, in combination with different drug encodings and machine learning models (Fig. 3). We found that Multilayer Perceptron (MLP) neural networks and Support Vector Machines (SVM) outperformed other models across MCC (Fig. 3), AUPRC, and balanced accuracy metrics (supplementary Fig. 2 and 3). Among the top 10 machine learning configurations out of the 40 evaluated, the MLP model appeared six times, while the SVM appeared four times. A similar trend, with MLP and SVM demonstrating superior performance, was observed when considering all species-drug pairs for MCC computation (supplementary Fig. 4).

Regarding dimensionality reduction of mass spectra, PCA and MAE demonstrated superior performance compared to mutual information and the absence of dimensionality reduction. Among the top 10 configurations out of the 40 evaluated, the MAE appeared four times, the PCA three times, mutual information two times and no preprocessing one time. Across all configurations, the mean MCC values for each dimensionality reduction method of the mass spectra were as follows: mutual information (0.35), no preprocessing (0.38), MAE (0.46) and PCA on MS (0.5).

For antifungal encoding, no clear performance differences were observed between MACCS fingerprints and Molformer SMILES encodings. Across all configurations, the mean MCC values were 0.42 for MACCS and 0.43 for Molformer SMILES encodings.

The highest performance among all tested configurations was achieved using a combination of PCA for mass spectra, Molformer SMILES antifungal encodings, and the MLP model, yielding an AUPRC of 0.77 and an MCC of 0.69 across the top 10 highest-performing species-drug pairs. The best-performing configuration used PCA with 99% variance retention and an MLP with two hidden layers (50 neurons each), a maximum of 1000 iterations, a tanh activation function, and a regularization alpha of 0.0001. This optimal machine learning configuration aligns with the overall performance patterns observed above. Therefore, we selected this configuration for the subsequent analyses.

### Unified species-and-drug-agnostic modeling matches species-drug-specific performance and improves applicability.

Incorporating drug fingerprints into the input features allows us to train a unified machine learning model that jointly predicts antifungal resistance across all species–drug combinations. This unified approach contrasts with alternative training schemes in which separate models are fitted either for each species, for each drug, or for each individual species–drug pair. While these more specialized models may better capture species-specific resistance mechanisms, they do not leverage shared information across different species and drugs. To systematically compare these training schemes, we evaluated their predictive performance using nested cross-validation on the training set, applying principal component analysis (PCA) to the mass spectra, MoLFormer SMILES-based drug embeddings, and a multilayer perceptron (MLP) classifier. To promote stable and meaningful evaluation, performance metrics were computed only for species–drug pairs with a class imbalance of at most 5% (ratio of resistant to susceptible observations) and at least two resistant samples.

Our results showed minimal performance differences between the different training schemes. The unified model trained jointly on all species–drug combinations achieved a mean MCC of 0.49 (±0.02), comparable to models trained separately for each species (0.46 ± 0.06), for each drug (0.47 ± 0.06), or for each individual species–drug pair (0.49 ± 0.03). While species-drug-specific models achieved higher performance on some clinically relevant pairs, for example, *C. tropicalis* with anidulafungin and *C. albicans* with fluconazole, they identified only one additional high-performing pair (MCC *\ge 0.04*) compared to the unified model. Conversely, the unified model offers broader applicability by enabling predictions for rare or low-sample-size species-drug pairs.

Additionally, we evaluated whether explicitly encoding antifungal drug class as an input feature improved predictive performance. Antifungal agents were grouped into azoles, echinocandins, and 5-fluorocytosine, and this categorical information was incorporated using one-hot encoding. Adding drug class as a separate input feature did not improve performance, likely because this information is already captured by the molecular representations used to encode the drugs.

Given the comparable predictive performance across training schemes and the broader coverage provided by a unified model trained on all species–drug combinations, we selected this jointly trained model for subsequent analyses. We refer to this approach as the *unified* model.

### Enhanced antifungal detection in *S. cerevisiae*, *C. albicans*, and *C. parapsilosis*

All subsequent results are based on analyses performed using the separate test set to ensure independent evaluation. To avoid skewed performance metrics, improve statistical validity and enhance generalizability, we report the unified model’s performance across the species-drug combinations where the maximum imbalance was 5% (either in resistance or susceptibility) and where at least two resistant samples were available, resulting in 12 pairs. AUPRC, MCC, balanced accuracy, number of true positives, true negatives, false positives and false negatives are reported in Table 2. Sensitivity, specificity, accuracy, very major error and major error are provided in Table 3.

The top-performing species-drug pairs in the test set are *S. cerevisiae*-itraconazole, which achieved perfect predictions, *C. albicans*-micafungin (MCC 0.79), *S. cerevisiae*-fluconazole (MCC 0.65), and *C. parapsilosis*-fluconazole (MCC 0.55). In terms of species, the unified model performed best on *S. cerevisiae*, achieving an average MCC of 0.83 across drugs, followed by *C. parapsilosis* (MCC 0.55) and *C. albicans* (MCC 0.49). Among antifungals, the unified model performed best with micafungin (average MCC 0.79 across species), followed by itraconazole (MCC 0.38) and fluconazole (0.24). These findings highlight the unified model’s ability to generalize across a diverse range of species and drugs.

We observed significant performance variability across species-drug pairs and even between different drugs for the same species. For instance, while the unified model achieved strong results for *C. albicans* with micafungin, it performed poorly when paired with posaconazole or voriconazole. Note that close results are obtained with the species-drug specific models (Supplementary Table 1). For *C. tropicalis*-anidulafungin, we obtained a negative MCC, indicating that the unified model performed worse than random guessing for this species-drug pair. This result may be due to the limited representation of *C. tropicalis*-anidulafungin samples in the training set, which could have prevented the unified model from learning meaningful resistance patterns specific to this combination. Alternatively, it may reflect intrinsic limitations in the biological signal captured by MALDI-TOF MS for detecting resistance to anidulafungin in *C. tropicalis*, suggesting that the spectral features available may not sufficiently differentiate between resistant and susceptible isolates for this particular case.

Because the proposed approach is designed to flag likely resistance to otherwise recommended therapy, false resistance alerts are particularly important, as they may lead to unnecessary changes in treatment. In contrast, when no resistance is predicted, the clinical workflow remains unchanged. We therefore prioritize high specificity and low major error rates, as these are the predictions most likely to influence treatment decisions. Thus, a low major error rate is prioritized even above minimizing very major errors.

Overall, our analysis demonstrated the unified model’s effectiveness in predicting antifungal resistance across various *Candida* and *Saccharomyces* species-drug pairs, with particularly strong performance for *S. cerevisiae* resistance to itraconazole and fluconazole, and *C. albicans* resistance to micafungin.

**Table 2:** Performance metrics on the test set, sorted by MCC, for the species-drug pairs with maximum imbalance 5% (either in resistance or susceptibility) and at least two resistant samples. *Imbalance* refers to the ratio of resistant to susceptible observations.

Species	Drug	MCC	AUPRC	AUROC	Imbalance	TP	TN	FP	FN
*Saccharomyces cerevisiae*	Itraconazole	1.00	1.00	1	0.63	5	3	0	0
*Candida albicans*	Micafungin	0.79	0.81	0.98	0.06	4	84	1	1
*Saccharomyces cerevisiae*	Fluconazole	0.65	0.73	0.5	0.75	6	1	1	0
*Candida parapsilosis*	Fluconazole	0.55	0.81	0.96	0.15	1	17	0	2
*Candida tropicalis*	Posaconazole	0.42	0.95	0.65	0.87	8	2	0	5
*Candida tropicalis*	Voriconazole	0.41	0.75	0.48	0.60	3	6	0	6
*Candida albicans*	Itraconazole	0.36	0.41	0.77	0.13	4	72	4	7
*Candida albicans*	Anidulafungin	0.32	0.54	0.70	0.26	10	56	9	13
*Candida tropicalis*	Itraconazole	0.26	0.87	0.59	0.73	6	3	1	5
*Candida glabrata*	Itraconazole	0.25	0.99	0.88	0.92	41	2	2	7
*Candida tropicalis*	Fluconazole	0.00	0.68	0.43	0.5	3	4	3	4
*Candida tropicalis*	Anidulafungin	-0.58	0.60	0.37	0.6	4	0	6	5

**Table 3:** Balanced accuracy, sensitivity, specificity, accuracy, very major error, and major error for species-drug combinations with more than 2 resistant samples in the test set and maximum imbalance 5% (either in resistance or susceptibility).

Species	Drug	Balanced acc	Sensitivity	Specificity	Accuracy	VME	ME
*Saccharomyces cerevisiae*	itraconazole	1.00	1.0000	1.0000	1.0000	0.0000	0.0000
*Candida albicans*	micafungin	0.89	0.8000	0.9882	0.9778	0.2000	0.0118
*Saccharomyces cerevisiae*	fluconazole	0.75	1.0000	0.5000	0.8750	0.0000	0.5000
*Candida parapsilosis*	fluconazole	0.67	0.3333	1.0000	0.9000	0.6667	0.0000
*Candida tropicalis*	posaconazole	0.81	0.6154	1.0000	0.6667	0.3846	0.0000
*Candida tropicalis*	voriconazole	0.67	0.3333	1.0000	0.6000	0.6667	0.0000
*Candida albicans*	itraconazole	0.66	0.3636	0.9474	0.8736	0.6364	0.0526
*Candida albicans*	anidulafungin	0.65	0.4348	0.8615	0.7500	0.5652	0.1385
*Candida tropicalis*	itraconazole	0.65	0.5455	0.7500	0.6000	0.4545	0.2500
*Candida glabrata*	itraconazole	0.68	0.8542	0.5000	0.8269	0.1458	0.5000
*Candida tropicalis*	fluconazole	0.5	0.4286	0.5714	0.5000	0.5714	0.4286
*Candida tropicalis*	anidulafungin	0.22	0.4444	0.0000	0.2667	0.5556	NaN

### Identification of spectral peaks driving antifungal resistance

The unified model performs best on *C. albicans*, *C. parapsilosis*, *C. tropicalis* and *S. cerevisiae*.

**Fig. 4:**
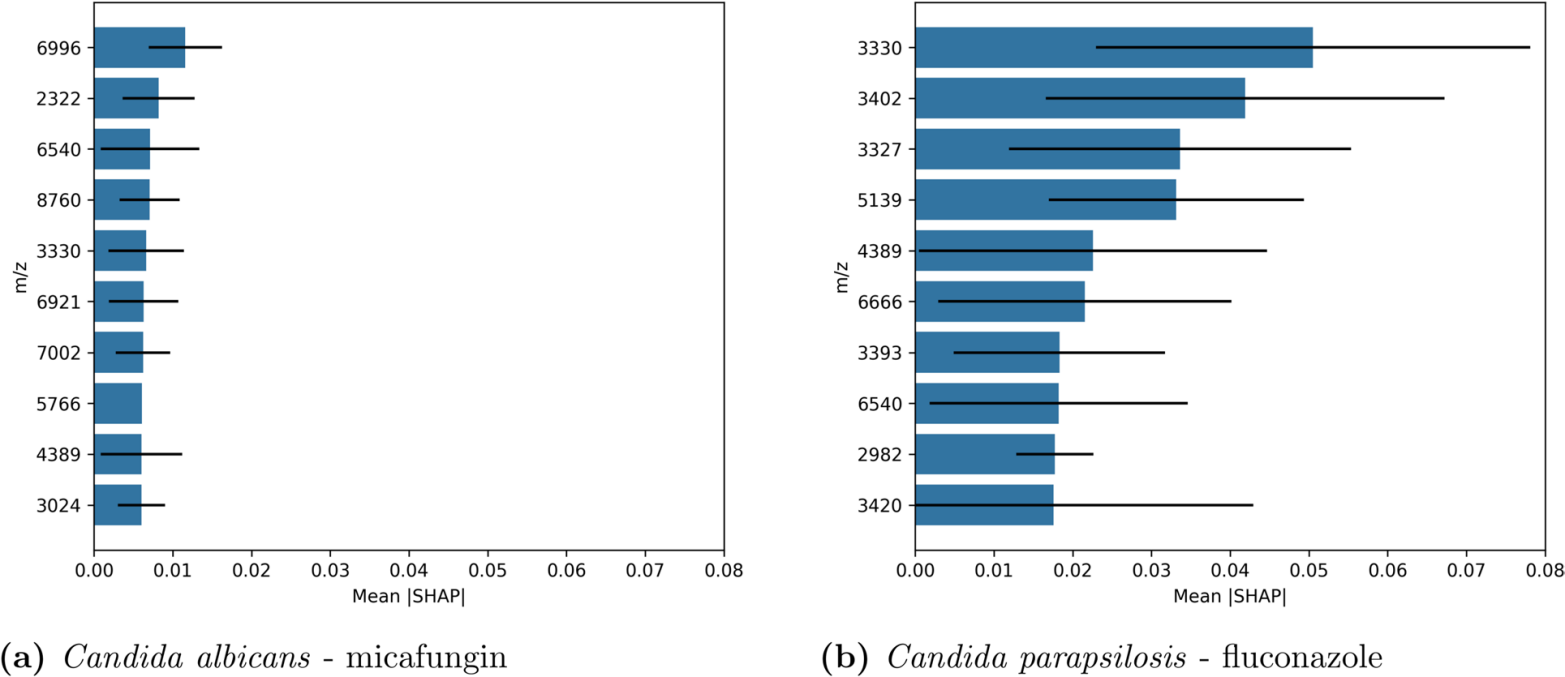
Comparison of SHAP-based feature importance for two species–drug pairs: *C. albicans*-micafungin (left) and *C. parapsilosis*-fluconazole (right). Bars represent the mean absolute SHAP values of the top 10 features, indicating their overall contribution to model predictions across bootstrap iterations. Error bars denote the standard deviation of SHAP values, reflecting variability and stability of feature contributions.

**Fig. 5:**
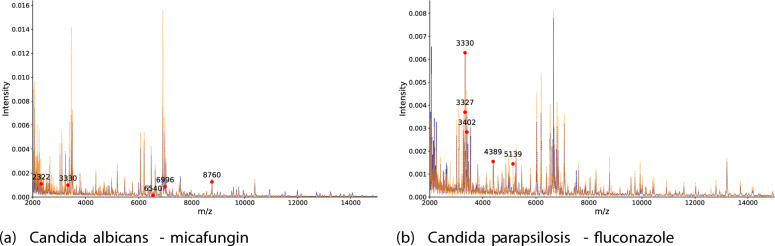
Average mass spectra for susceptible (blue) and resistant (dashed orange) pathogens. Red dots indicate the top-5 peaks that contribute most to AMR prediction (“Model properties and interpretability”).

For *C. albicans* treated with Micafungin (Fig. 4a), the overall magnitude of feature contributions was relatively low and tightly clustered, with all top features exhibiting small mean absolute SHAP values and limited variability. This suggests that the model relies on a diffuse set of weak signals, with no single feature exerting a dominant influence on the prediction. The relatively narrow error bars further indicate stable feature contributions across bootstrap iterations, pointing to consistent but modest predictive signals. Figure 5a shows the difference in average mass spectra between *C. albicans* isolates that are resistant (blue) and susceptible (orange) to micafungin. The SHAP values, which indicate the contribution of each m/z feature to the classification, were computed from 90 observations. Among these, features corresponding to m/z 2322, 3330, 6540, 6996 and 8760 (*± 1*), emerged as the most influential in distinguishing resistant from susceptible isolates. These peaks may represent potential biomarkers linked to micafungin resistance in *C. albicans*.

The model for *C. parapsilosis* treated with Fluconazole demonstrated substantially higher feature importance values and greater variability. Several features (e.g., m/z 3330, 3402, 3327) showed markedly larger mean SHAP magnitudes, indicating a stronger and more concentrated dependence on specific predictors. The wider error bars associated with these features reflect increased variability in their contributions across bootstrap samples, suggesting that while these features are influential, their effects may be sensitive to data perturbations. Figure 5b illustrates the average mass spectra differences for *C. parapsilosis* isolates resistant (blue) and susceptible (orange) to fluconazole. SHAP values were computed using 20 samples. The five most discriminative m/z features identified in this analysis were 3327, 3330, 3402, 4389, and 5139 (*± 1*).

Supplementary Figure 5 illustrates the average mass spectra differences for (a) *S. cerevisiae* isolates resistant and susceptible to fluconazole, and (b) *C. tropicalis* to posaconazole. Notably, across *C. albicans* treated with Micafungin, *S. cerevisiae* treated with Fluconazole, and *C. tropicalis* treated with Posaconazole, the feature corresponding to m/z 6996 consistently emerged as the most influential predictor. This suggests the presence of a potentially conserved signal contributing to antifungal response across multiple species–drug combinations. However, beyond this shared feature, the feature importance profiles diverged substantially, indicating distinct predictive patterns specific to each species–drug context. Overall, this approach has enabled the identification of specific mass spectral biomarkers associated with antifungal resistance.

### Estimated clinical impact: reduced selection of antifungals associated with resistance

In clinical practice, not all predictions hold the same value, as certain antifungal drugs are rarely used for particular species. To evaluate the utility of our ML approach, we determine the frequency at which a recommended antifungals would have failed according to an antifungal susceptibility tests in the DRIAMS dataset. We determine the percentage of incorrect recommendations using species-based guidelines, which define the expected activity of antifungal agents against specific fungal species. For each species, the guidelines indicate whether a given agent is considered: *generally effective and recommended for therapy*, *generally effective*, *variably effective*, associated with a *high probability of resistance*, or if *no or insufficient data* are available. We limit our analyses to the 7 drugs and 6 species present in both the DRIAMS data and for which we also have guideline recommendations (184 samples in the test set). These include only *Candida* species. For many species, multiple antifungals are equally recommended as the top choices (i.e., *generally effective and recommended for therapy*). Since no additional patient information is available in the dataset, we hypothesize that physicians would choose randomly among them. For example, if a hospital recommends three drugs equally for a species and two of them are effective according to the susceptibility tests, we consider the hospital’s recommendation to be 66% correct for the pathogen investigated. In the test set, using the hospital species-based recommendations, 9% of the pathogens have at least a 25% risk of being resistant to the received drug according to susceptibility tests. These cases involved *C. albicans*, *C. dubliniensis*, *C. glabrata*, *C. parapsilosis*, and *C. tropicalis*. The antifungals associated with these cases are fluconazole, anidulafungin, and caspofungin. Importantly, antifungal susceptibility tests indicated that an antifungal associated with susceptibility could have been provided in 100% of these cases.

We applied the unified model (“Combination of dimensionality reduction and neural networks yield the highest antifungal prediction performance”) to the 9% of pathogens in the test set with at least a 25% risk of resistance. These cases are particularly challenging because species-based guidelines suggest antifungals that are generally expected to be effective but prove ineffective for the specific pathogen according to susceptibility testing. Within this subset, resistance was correctly identified in 29% of cases. This indicates that, among patients for whom species-based guidelines recommend antifungals to which their pathogens are in fact resistant (based on susceptibility tests), the approach could have flagged resistance earlier than conventional susceptibility testing in approximately 3 out of 10 cases (29%).

We also assessed how often the unified model incorrectly predicted resistance for pathogens that were in fact susceptible, and the potential clinical impact of such errors. Resistance was incorrectly predicted in 3% of cases. However, in all instances, at least one alternative top-ranked antifungal was correctly predicted as susceptible, ensuring that physicians could still select an effective treatment option.

## Discussion

To assess the potential for early detection of antifungal resistance using MALDI-TOF MS, we explored a range of machine learning architectures and compared findings to phenotypical results. Our experiments showed that using more complex models to encode drugs molecular information did not consistently lead to better performance. While the top-performing configuration incorporated the transformer-based Molformer-derived drug embeddings, overall results were comparable between the Molformer encodings and simpler MACCS fingerprints. Also, the more complex dimensionality reduction method for mass spectra, Masked Autoencoder (MAE), did not yield performance gains compared to simpler approaches like PCA. Several factors could explain why these more sophisticated methods failed to outperform simpler alternatives. One reason can be overfitting, as deep learning models like MAE typically require large datasets to generalize effectively. In our case, the base dataset (without dimensionality reduction) comprises 3,046 observations, with 6,000 mass spectra features and between 166 and 768 drug-related features. Additionally, in this project, we used Molformer model checkpoints pre-trained on only 10% of a larger dataset—approximately 100 million molecules, with 10% sourced from ZINC and another 10% from PubChem, originally used for training Molformer-XL. Gaining access to a Molformer model trained on the full dataset, rather than just a 10% subset, could enhance performance by providing richer and more comprehensive molecular encodings.

A key limitation of this study is therefore the relatively limited dataset size and the constrained nature of external validation. Although we evaluated the model on an independent dataset (Supplementary Section 3), the external dataset was small and lacked sufficient diversity in outcomes, preventing a comprehensive assessment of generalizability. Consequently, external validation results should be interpreted with caution, and future work should include validation on larger, more heterogeneous external cohorts with balanced representation of resistant and susceptible isolates to more reliably assess generalizability.

We observed substantial variability in predictive performance across species–drug pairs, which cannot be fully explained by differences in sample size or class imbalance alone. For instance, some combinations with relatively limited data (e.g., *S. cerevisiae*-itraconazole) or higher imbalance (e.g., *C. albicans*-micafungin) still achieved strong performance, whereas others with larger sample sizes (e.g., *C. albicans*-anidulafungin) or more balanced class distributions (e.g., *C. tropicalis*-fluconazole) performed comparatively poorly. Several additional factors may contribute to the observed variability in performance. First, the variability likely reflects differences in the biological signal captured by MALDI-TOF mass spectra. This signal depends on the underlying resistance mechanisms, as only those that induce consistent and measurable proteomic changes can be reliably detected. Mechanisms involving altered protein expression, such as efflux pump overexpression or metabolic adaptations, may generate more pronounced and reproducible spectral signatures that facilitate prediction. In contrast, subtle molecular changes may not produce detectable spectral differences, limiting model performance. In addition, these changes must occur within the low-molecular-weight protein range detectable by MALDI-TOF, as alterations outside this range will not be captured in the spectra. Second, spectral profiles may exhibit either high similarity or substantial heterogeneity within a species, reducing separability between resistant and susceptible isolates. Finally, technical factors related to MALDI-TOF acquisition and preprocessing, including signal-to-noise ratio, peak alignment, and binning, may further influence the detectability of relevant features across species–drug combinations.

Most MALDI-TOF-based susceptibility testing protocols differ from our approach as they require an incubation period of at least three hours with varying antifungal concentrations. To assess the effectiveness of our direct prediction approach, we compared its performance with results reported for these incubation-based methods. Focusing on species-drug pairs in Table 2, we identified published performance metrics for three pairs. For *C. tropicalis*-fluconazole, Saracli et al.^10^ observed a very major error (VME; false susceptible/total resistant) of 0.14 (1/7) and a major error (ME; false resistant/total susceptible) of 0.43 (12/28). Our model produced a higher VME of 0.57 (4/7) and a close ME of 0.42 (3/7). For *C. tropicalis*-posaconazole, Saraclı et al. reported a VME of 0.5 (2/4) and a ME of 0.18 (6/33), while our model yielded improved VME of 0.38 (5/13) and ME of 0 (0/2). For *C. tropicalis*-voriconazole, Saracli et al. found a VME of 0.4 (2/5) and an ME of 0.36 (10/28), whereas our model produced a higher VME of 0.67 (6/9) but a ME of 0 (0/3). Thus, our method consistently achieves a lower major error rate, demonstrating greater precision in avoiding false resistance predictions. However, it tends to underpredict resistance, resulting in higher very major errors in some cases. This balance aligns with our *warning detection* approach: if resistance goes undetected, the clinician’s workflow remains unchanged. However, when the model flags resistance, it is critical, as it may change treatment decisions. Compared to MALDI-TOF-based susceptibility testing, a key advantage of our approach is that it eliminates the need for additional laboratory experiments, reducing both processing time and costs, making it more suitable for routine clinical use.

The proposed AI-assisted “warning system” extends routine MALDI-TOF–based diagnostics by providing early predictions of antifungal resistance at the time of species identification. In current clinical practice, patients with suspected fungal infections are typically started on empiric antifungal therapy while diagnostics are ongoing. MALDI-TOF enables rapid species identification, but isolate-specific antifungal susceptibility testing requires an additional 24–48 hours, delaying precise treatment adjustments. To bridge this gap, our approach leverages MALDI-TOF spectra of the individual patient isolate to estimate resistance probabilities immediately after species identification. These predictions can be reported alongside the identification result, enabling earlier, evidence-based decision support. Concretely, once a MALDI-TOF spectrum is acquired and species identification is completed, the model generates a resistance probability for each antifungal agent. If the predicted probability of resistance to a commonly recommended antifungal exceeds a predefined threshold, a warning can be included in the laboratory report, indicating that the standard first-line antifungal therapy may be ineffective. For example, if *C. albicans* is identified and resistance to fluconazole is predicted, an alert would be issued at the time of identification, allowing earlier consideration of alternative treatment options before AFST results become available, and potentially prompting targeted confirmatory testing where specific resistance mechanisms can be further validated.

Integrating early antifungal resistance prediction based on MALDI-TOF mass spectra into routine hospital diagnostics presents several practical challenges. Standardizing data preprocessing across different instruments, laboratory protocols, and software environments is essential, as is ensuring robust model validation across diverse patient populations, hospitals, geographic regions, and time periods^35^. Seamless integration into laboratory information systems will also be required, ideally through intuitive, clinician-friendly interfaces. In addition, regulatory approval, clinician trust in AI-assisted tools, and the interpretability of model outputs will be critical for successful clinical adoption.

These include standardizing data preprocessing across different MALDI-TOF instruments, laboratory protocols, and software environments, as well as ensuring robust model validation across diverse patient populations, hospitals, geographic regions, and time periods^35^. Seamless integration into laboratory information systems is also essential, ideally with an intuitive, clinician-friendly dashboard that fits naturally into existing diagnostic workflows. Additionally, regulatory approval, clinician trust in AI-assisted tools, and clear interpretability of model outputs will be critical for adoption.

Future evaluations will be necessary to further validate our findings and assess the real-world impact of our approach. Since our results are based on data from the University Hospital Basel, validating the model’s performance on an external dataset would strengthen confidence in its generalizability. Additionally, the spectral m/z units identified as contributors to resistance should also be experimentally validated, for instance, by integrating sequencing data to confirm their biological relevance. This is particularly important given that resistance mechanisms are often complex and context-dependent, requiring validation beyond computational inference. Incorporating genomic data in future work would allow for peak annotation and biological interpretation. Moreover, our approach uses binary resistance/susceptibility labels, whereas working with continuous outcomes, such as minimum inhibitory concentrations (MICs), could provide a more nuanced and informative representation of antifungal resistance. Finally, conducting a prospective multi-center randomized controlled trial will help validate our findings and assess whether our model would influenced treatment decisions in a clinical setting, including its potential impact on antifungal de-escalation strategies. These future steps will be essential in translating our findings into practical clinical applications.

## Supplementary Information

Supplementary Information.

## Data Availability

The MALDI-TOF spectra used in this project can be obtained by downloading the DRIAMS dataset from https://doi.org/10.5061/dryad.bzkh1899q . The code necessary to reproduce this article’s results and analyses is available on GitHub at https://github.com/DianeDuroux/AntifungalResistancePrediction/
