# Supplementary material for: Early antifungal resistance prediction based on MALDI-TOF mass spectrometry and machine learning
Source: Sci Rep. 2026 Jun 2;16:25150. doi: 10.1038/s41598-026-53519-y (PMC13470391; doi:10.1038/s41598-026-53519-y)
Supplement: Supplementary file 1 [file 41598_2026_53519_MOESM1_ESM.pdf]

Supplementary

Early antifungal resistance prediction based on MALDI-TOF mass spectrometry and machine learning

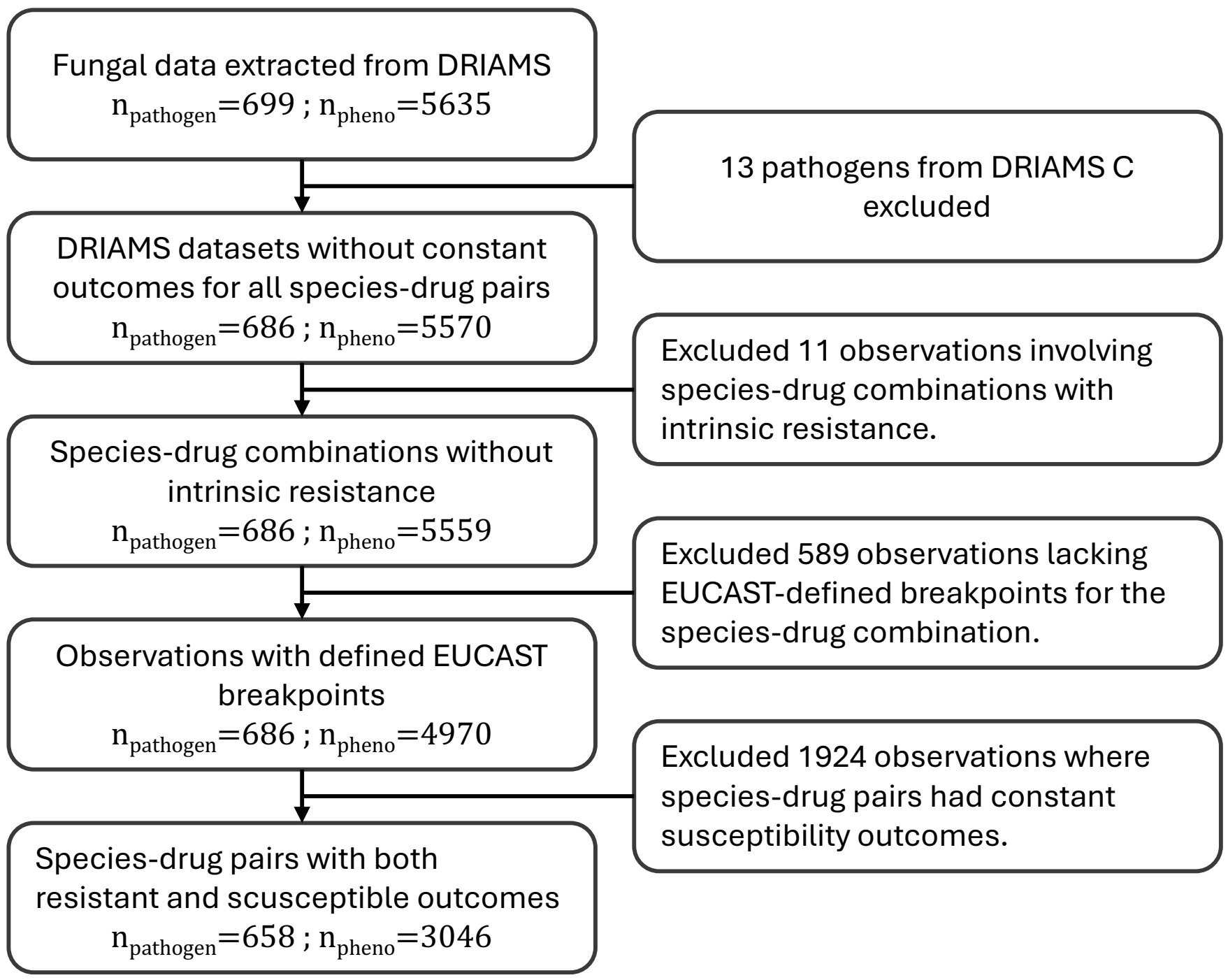

Fig. 1: Overview of data exclusion steps during preprocessing. $n_{pathogen}$ is the number of unique pathogen mass spectra in the dataset and n_{pheno} is the number of associated phenotypic antifungal resistance results. An observation is a pathogen-drug pair.

1 Machine learning approaches parameters

Logistic Regression (LR) was implemented with `max_iter` set to 2000 to ensure convergence during optimization. We evaluated the model using C values of 0.1, 1, and 10, with both L1 and L2 regularization penalties.

Support Vector Machines (SVM) with a Radial Basis Function (RBF) kernel were employed, using C values of 0.1, 1, and 10 to balance error minimization and model complexity. The `probability` parameter was set to `True` to enable class probability estimation.

For Random Forest (RF), we configured the model with 50, 100, and 200 trees (`n_estimators`) and tested maximum depths of None, 10, and 20 (`max_depth`). All other parameters were kept at

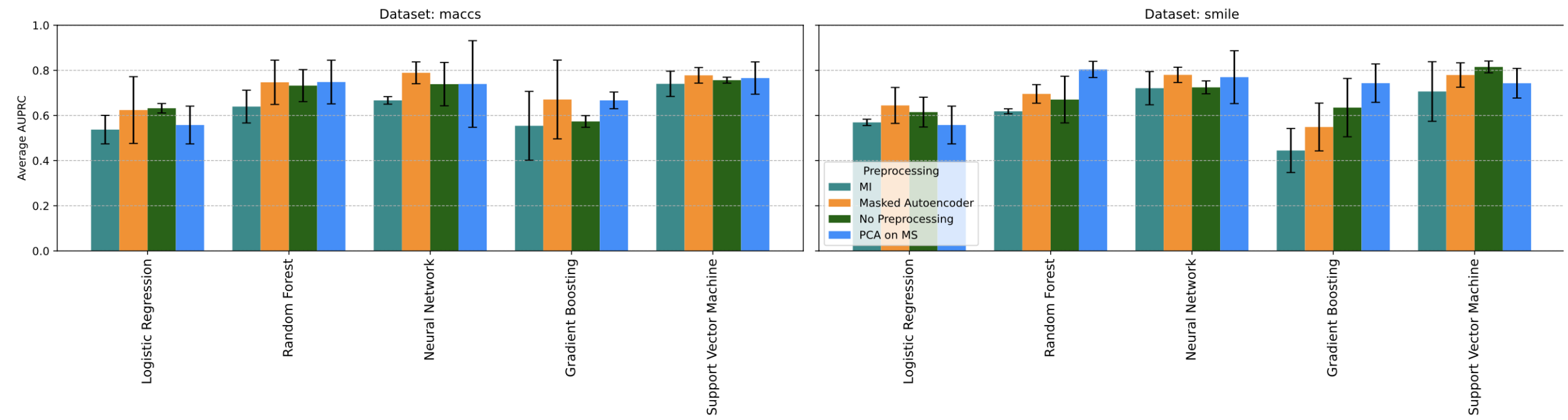

Fig. 2: AUPCR across pre-processing techniques and models for the top 10 highest-performing species-drug pairs. Error bars show the standard deviation computed via the 3-fold cross validation.

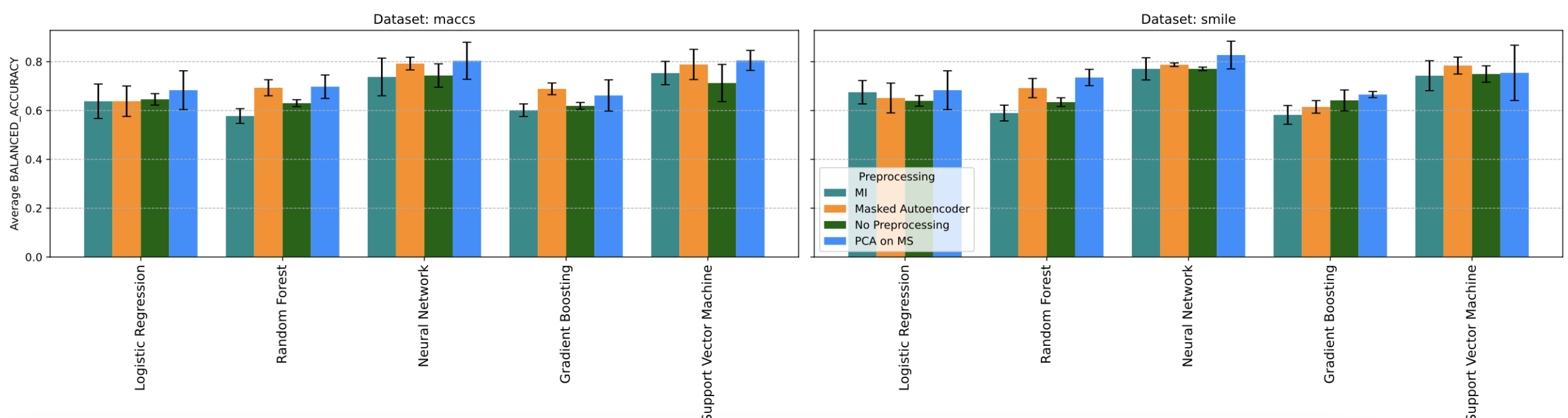

Fig. 3: Balanced accuracy across pre-processing techniques and models for the top 10 highest-performing species-drug pairs. Error bars show the standard deviation computed via the 3-fold cross validation.

their default values as specified in the `RandomForestClassifier` function in Python.

Gradient Boosting Classifier (GBC) was set with 50, 100, and 200 boosting stages (`n_estimators`), and a learning rate of 0.01, 0.1, 0.2.

Multilayer Perceptron (MLP) was implemented with hidden layers of sizes (100,), and (50, 50), a maximum of 1000 iterations (`max_iter`),alpha of 0.0001 and 0.001, and the ReLU and tanh activation function.

All models were implemented using the scikit-learn package (Pedregosa et al., 2011).

2 Performance of the species-drug specific models

We analyzed the model's performance across various species-drug combinations using predictions from Set 2 to evaluate its effectiveness in different contexts. The model performed best on *Candida*

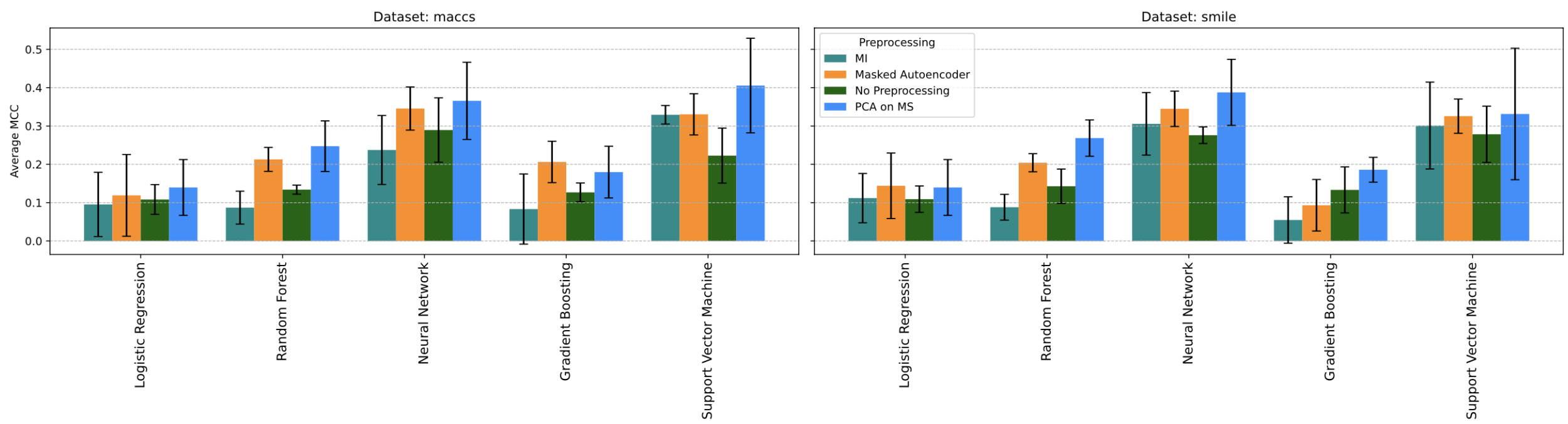

Fig. 4: MCC across pre-processing techniques and models for all species-drug pairs. Error bars show the standard deviation computed via the 3-fold cross validation.

parapsilosis, achieving an average MCC of 0.84 across drugs, followed by *Saccharomyces cerevisiae* (MCC 0.83) and *Candida albicans* (MCC 0.29). Similarly, among antifungals, the model performed best with micafungin (average MCC 0.89 across species), followed by 5-fluorocytosine (MCC 0.34) and caspofungin (MCC 0.33). Note that for five *Candida* species, the models were not directly trained on caspofungin. Instead, following EUCAST guidelines, isolates that were susceptible to anidulafungin as well as micafungin were considered susceptible to caspofungin. Overall, these findings highlight the model's ability to generalize across a diverse range of species and drugs. However, we observed significant performance variability across species-drug pairs and even between different drugs for the same species. For instance, while the model achieved strong results for *Candida albicans* with micafungin, it performed poorly when paired with posaconazole or voriconazole. The top-performing species-drug pairs in Set 2 are *Saccharomyces cerevisiae*–itraconazole, which achieved perfect predictions, followed by *Candida albicans*–micafungin (MCC 0.89), *Candida parapsilosis*–fluconazole (MCC 0.84), *Candida tropicalis*–5-fluorocytosine (MCC 0.68)*Saccharomyces cerevisiae*–fluconazole (MCC 0.65).

Species	Drug	AUPRC	MCC	Balanced acc	Imbalance	TP	TN	FP	FN
saccharomyces cerevisiae	itraconazole	1.00	1.00	1.00	0.63	5	3	0	0
Candida albicans	micafungin	0.86	0.89	0.90	0.06	4	85	0	1
Candida parapsilosis	fluconazole	1.00	0.84	0.97	0.15	3	16	1	0
Candida tropicalis	5-fluorocytosine	0.58	0.68	0.75	0.13	1	13	0	1
saccharomyces cerevisiae	fluconazole	0.82	0.66	0.75	0.75	6	1	1	0
Candida tropicalis	posaconazole	0.98	0.48	0.85	0.87	9	2	0	4
Candida albicans	anidulafungin	0.60	0.40	0.69	0.26	12	56	9	11
Candida albicans	itraconazole	0.42	0.35	0.68	0.13	5	69	7	6
Candida albicans	itraconazole	0.97	0.14	0.62	0.92	35	2	2	13
Candida glabrata	itraconazole	0.84	0.04	0.52	0.73	6	2	2	5

Table 1: Performance metrics for the top-10 species-drug pairs, sorted by MCC. For caspofungin, susceptibility is inferred using both anidulafungin and micafungin (logical AND).

3 External validation

Our primary analyses were conducted on the DRIAMS dataset; however, the DRIAMS-C subset (Canton Hospital Aarau) was excluded from model development because it contains only susceptible isolates and therefore lacks the outcome variability required for supervised learning. To nevertheless provide an element of external validation, we evaluated the trained model on DRIAMS-C as an independent test set. Due to the absence of resistant isolates in this dataset, evaluation is restricted to assessing false positive rates, and standard performance metrics such as MCC, AUROC, or AUPRC cannot be meaningfully computed. For species–drug pairs represented in both the training data (DRIAMS-A) and DRIAMS-C, the model predicted all samples as susceptible, consistent with the observed labels. While this yields perfect apparent sensitivity, these results must be interpreted cautiously given the very small sample sizes—*Candida parapsilosis*–fluconazole (n=3), *Candida tropicalis*–fluconazole (n=2), and *Candida tropicalis*–voriconazole (n=2), and the absence of resistant cases.

These results highlight that while the model shows some ability to generalize to external data, the evidence remains limited. The external dataset is small and lack sufficient diversity in outcomes, which restricts robust performance assessment. Future work should include validation on larger, more heterogeneous external cohorts with balanced representation of resistant and susceptible isolates to more reliably assess generalizability.

4 Identification of spectral peaks driving antifungal resistance

For *S. cerevisiae*–Fluconazole, the model exhibited a broad distribution of influential features with relatively high mean SHAP values, particularly for m/z 6996, 3009, and 5781. This suggests that predictions are driven by multiple strong contributors, indicating a more complex or distributed decision structure. The relatively wide error bars associated with these features further indicate variability in their contributions across bootstrap iterations, reflecting sensitivity to data perturbations. The SHAP values were computed from 8 observations. The 5 top mass spectra features differentiating between cases and controls are 3009, 5778, 5781, 6021, and 6996 m/z (± 1).

In contrast, the *C. tropicalis*–Posaconazole model displayed generally lower SHAP magnitudes and a more gradual decline in feature importance beyond the top-ranked predictors. While 6996 and 7002 were the most influential features, the overall effect sizes were smaller and more homogeneous, suggesting a less sharply defined set of dominant predictors. The narrower range of SHAP values implies that the model relies on a more balanced combination of features rather than a few highly dominant ones. The SHAP values were computed from 15 observations. The 5 top mass spectra peaks differentiating between cases and controls are 3501, 6996, 6999, 7002, 7212 m/z (± 1).

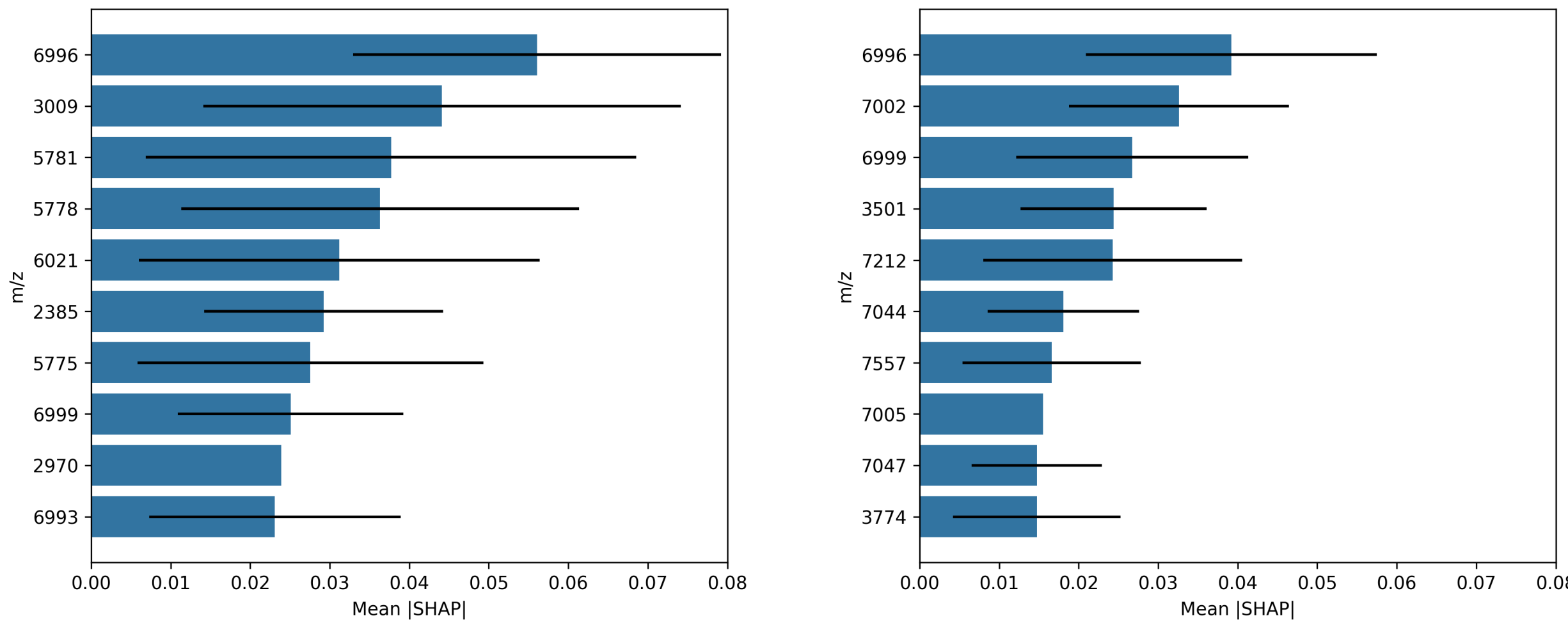

(a) *Saccharomyces cerevisiae* - fluconazole

(b) *Candida tropicalis* - posaconazole

Fig. 5: Comparison of SHAP-based feature importance for two species–drug pairs: *Saccharomyces cerevisiae*–Fluconazole (left) and *Candida tropicalis*–Posaconazole (right). Bars represent the mean absolute SHAP values of the top 10 features, indicating their overall contribution to model predictions across bootstrap iterations. Error bars denote the standard deviation of SHAP values, reflecting variability and stability of feature contributions.

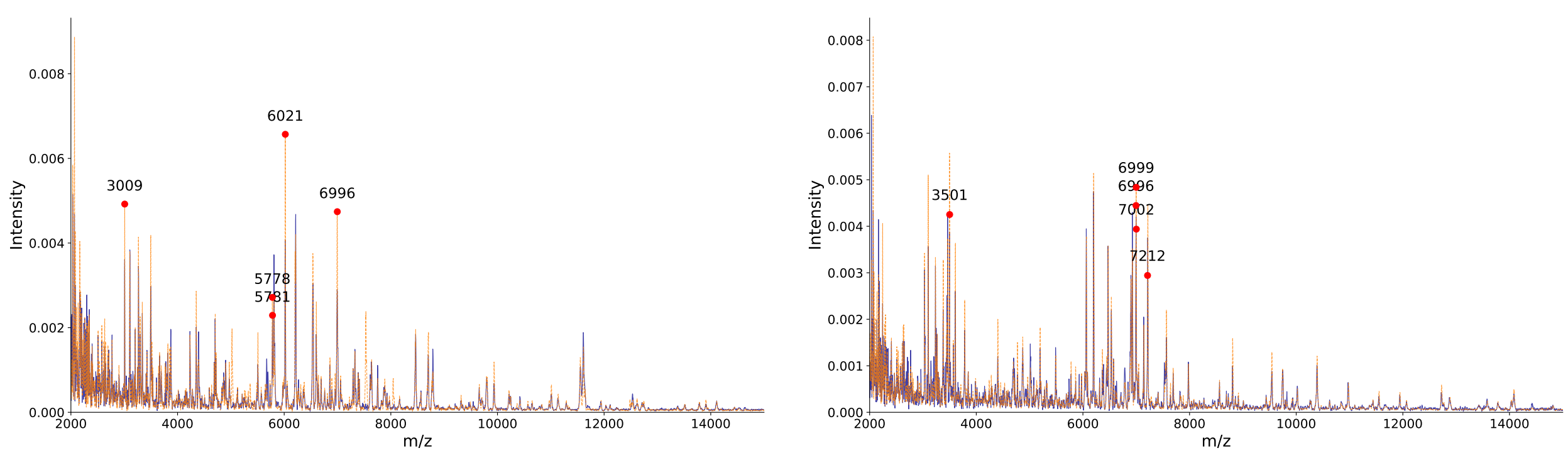

(a) *Saccharomyces cerevisiae* - fluconazole

(b) *Candida tropicalis*- posaconazole

Fig. 6: Average mass spectra for susceptible (blue) and resistant (dashed & orange) pathogens. The peaks contributing the most to AMR prediction are annotated with red dots.

5 Fungal Isolate Identification and Antifungal Susceptibility Testing

Fungal isolates were identified using the Microflex Biotyper database integrated within the flexControl software (Bruker Daltonics flexControl v3.4). Subsequently, minimum inhibitory concentrations for individual strains were determined using the YeastOne assay, a standardized microdilution method. Results were interpreted according to clinical breakpoints defined by CLSI and EUCAST guidelines, enabling classification of isolates based on their susceptibility profiles.
